# *Viscum album* L. mother tinctures modulate Na^+^/K^+^ ATPase activity and expression, and promote endothelium-dependent vasodilation via SK channel and nitric oxide signalling

**DOI:** 10.3389/fphys.2025.1736143

**Published:** 2026-02-02

**Authors:** Rodrigo dos Santos Pinto Duarte, Michelle Nonato de Oliveira Melo, João V. C. Batista, Giovanna Gomes Martins, Adriana Passos Oliveira, Rosilane Taveira-da-Silva, Maria Luiza Fidelis da Silva, Rafael H. F. Valverde, Marcelo Einicker-Lamas, Arquimedes Gasparotto Junior, Stephan Baumgartner, Carla Holandino

**Affiliations:** 1 Multidisciplinary Laboratory of Pharmaceutical Sciences, Faculty of Pharmacy, Universidade Federal do Rio de Janeiro, Rio de Janeiro, Brazil; 2 Society for Cancer Research, Hiscia Institute, Arlesheim, Switzerland; 3 Instituto de Biofísica Carlos Chagas Filho, Universidade Federal do Rio de Janeiro, Rio de Janeiro, Brazil; 4 Laboratory of Cardiovascular Pharmacology, Faculty of Health Sciences, Universidade Federal da Grande Dourados, Dourados, Mato Grosso do Sul, Brazil; 5 Institute for Integrative Medicine, University of Witten/Herdecke, Herdecke, Germany; 6 Institute for Complementary and Integrative Medicine, University of Bern, Bern, Switzerland

**Keywords:** hypertension, kidney enzymes, mother tinctures, vasodilation, *Viscum album*

## Abstract

**Introduction:**

Ethanolic extracts of *Viscum album* L. (European mistletoe), known as mother tinctures (VAMTs), have been used in traditional medicine to treat hypertension and cardiovascular diseases. However, the underlying biological mechanisms remain poorly characterized.

**Methods:**

This study investigated the cytotoxic, and cardiovascular effects of ten VAMTs prepared from fresh plants harvested in summer and winter. The extracts were derived from three subspecies (*V. album subsp. album, abietis, and austriacum*) growing five distinct host tree species. Cytotoxicity and oxidative stress were assessed *in vitro*, while the effects on Na^+^/K^+^-ATPase activity and expression were evaluated in porcine renal proximal tubular cells. Additionally, vascular effects were investigated in perfused mesenteric vascular beds of spontaneously hypertensive rats.

**Results and Discussion:**

Our data demonstrated that VAMTs exhibit no cytotoxic effects, except for the VAMT derived from *Quercus petraea*, and do not induce oxidative stress in *in vitro* assays. Regarding molecular targets, distinct profiles were observed: the VAMT derived from A. alba (summer harvest) reduced Na^+^/K^+^- ATPase expression and activity in the cellular model, whereas all winter VAMTs reduced only the expression of this enzyme. The winter VAMT from *P. sylvestris* (VAMT PW) elicited vasodilation in resistance arteries from hypertensive rats. This vasodilatory effect appears to be mediated by the nitric oxide/soluble guanylate cyclase/cyclic guanosine monophosphate signalling pathway and the subsequent activation of small conductance calcium-activated potassium channels. These data support the efficacy of VAMT PW and emphasise the impact of the host tree and season for optimizing antihypertensive potential of *V. album* samples in the context of traditional mistletoe medicine.

## 1 Introduction

Hypertension is a chronic cardiovascular condition with multifactorial aetiology, capable of causing systemic and vascular damage (Stanaway et al., 2018). It is defined as sustained blood pressure at or above 140/90 mmHg. Globally, hypertension affects one in three adults, with its prevalence doubling in the Americas from 650 million cases in 1990 to 1.3 billion in 2019. More than three-quarters of these cases occur in low- and middle-income countries (PAHO, 2021; PAHO, 2022). Furthermore, hypertension is the most common chronic disease worldwide and remains a leading cause of mortality and disability (Mills et al., 2020).

The current treatment of hypertension involves the use of various drug classes, including diuretics, sympatholytic agents, direct vasodilators, renin-angiotensin system inhibitors, and calcium channel blockers (Rang et al., 2007; Guyton and Hall, 2006). However, treatment often requires a combination of drugs from different classes, increasing the risk of side effects. To mitigate these adverse effects and improve patient adherence, there is growing interest in the development of plant-derived products (herbal medicine). These herbal compounds are increasingly studied due to their lower incidence of side effects, in addition to being safe and effective, offering a promising alternative to conventional treatments (Newman and Cragg, 2016; Ofem et al., 2007; Poruthukaren et al., 2014).

The species *Viscum album *L. (*V. album*) is a semiparasitic plant belonging to the Santalaceae family, native to Europe, Asia and northeast Africa (Melo et al., 2023; WFO, 2025). European *V. album* is a dioecious species, with female flowers that produce fruits ripening during the winter season. Commonly known as mistletoe, this plant has a long history of use in traditional medicine for the treatment of different pathologies, including cancer and cardiovascular diseases (Jadhav N. et al., 2010; Melo et al., 2023; Olas, 2024; Poruthukaren et al., 2014).

The composition of preparations derived from mistletoe varies significantly based on factors such as the host tree, the season of harvest, the collection site, and the solvent used for extraction (Jäger et al., 2021; Melo et al., 2022). The main subspecies of European *V. album* L. include *V. album* subsp*. album*, which grows on deciduous trees, such as *Malus domestica*, *Quercus* sp. and *Ulmus carpinifolia*; *V. album* subsp*. austriacum* grows on *Pinus sylvestris;* and *V. album* subsp*. abietis* which grows on *Abies alba*. The host trees confer unique qualitative and quantitative characteristics to *V. album* extracts and consequently influence their therapeutic properties (Holandino et al., 2020; Melo et al., 2022; Song et al., 2021).

Several studies have reported that mistletoe species harvested from various host trees exhibit antihypertensive activity (Anne et al., 2011; Bachhav et al., 2012; Deliorman et al., 2000; Dobrecky et al., 2022; Engelbrecht, 2006; Jadhav R. B. et al., 2010; Khan et al., 2016; Ofem et al., 2007; 2009; Poruthukaren et al., 2014; Radenkovic et al., 2009; Rodríguez-Cruz et al., 2003; Sepúlveda-Orellana et al., 2025; Suveren et al., 2017; Tenorio et al., 2005). However, despite significant effects observed in *in vitro* and *in vivo* models, their underlying mechanisms of action remain poorly understood. Given the global burden of hypertension, reactive oxygen species (ROS) have been identified as primary drivers of endothelial dysfunction. Concurrently, renal Na^+^/K^+^ ATPase has emerged as a key regulator of tubular sodium reabsorption, extracellular fluid volume, and long-term blood pressure control (McDougall and Yates, 1998). Furthermore, vascular Na^+^/K^+^ ATPase plays a pivotal role in controlling arterial tone. By signalling through the proto-oncogene tyrosine-protein kinase Src, it contributes to the modulation of Ca^2+^ sensitivity in vascular smooth muscle cells (Staehr et al., 2023). Therefore, the present study aimed to characterize the pharmacological effects of *V. album* mother tinctures (VAMTs), focusing on their ability to modulate ROS concentration and Na^+^/K^+^ ATPase expression and activity in a porcine proximal tubule epithelial cell line, as well as to elucidate the molecular mechanisms underlying vasodilation in the perfused mesenteric vascular bed (MVB) of spontaneously hypertensive rats (SHR).

## 2 Materials and methods

### 2.1 *Viscum album* L. Samples

Winter and summer *V. album* mother tinctures from five host trees (*M. domestica*, *Quercus petraea*, *U. carpinifolia*, *P. sylvestris* and *A. alba*) were harvested at Rütti and Disli, Switzerland. The plant material was previously identified by Dr. Marcelo Guerra Santes (Universidade Estadual do Rio de Janeiro), and voucher specimens were deposited at the Herbarium of the Faculdade de Formação de Professores, Universidade Estadual do Rio de Janeiro, Brazil (Holandino et al., 2020). Following summer and winter harvests, the plants were processed by hydroethanolic maceration, according to pharmacopeial methodologies (ANSM, 2010; Brasil, 2011).

The leaves and stems were cut into 5 cm fragments and mixed with ripe (winter) or unripe (summer) berries. The plant material underwent static maceration for 3 weeks at room temperature. The solvent was a hydroethanolic solution of 70%–90% (v/v), standardized to a solvent-to-plant ratio of 10%, based on the dry residue of the fresh plant (Brasil, 2011). After maceration, the resulting mother tinctures were filtered and stored at 20 °C ± 4 °C. The final alcohol content was 60% (v/v) for all VAMTs (Holandino et al., 2020). The quantitative details relating to each VAMT preparation can be found in the Supplementary Material.

For experiments on the peripheral vascular resistance of hypertensive rats, the VAMTs were evaporated at 40 °C in a water bath (Büchi, Flawil, Switzerland) under vacuum (V-700 vacuum pump, Büchi, Flawil, Switzerland). The residue obtained was frozen overnight at −80 °C and subsequently lyophilised (Christ Beta 2-8 LD) for 72 h, at −43 °C/0.09 mbar. This procedure resulted in a dry VAMT sample. The freeze-dried winter and summer VAMTs samples from *A. alba*, *M. domestica* and *P. sylvestris* were administered at specific doses, as described in Section 2.9.2. These dried extracts showed the approximately yield of 12, 17, 7.8, 12.5, 10.4, 9.4% for *A. alba*, *M. domestica* and *P. sylvestris* from summer and winter seasons, respectively.

### 2.2 HPTLC analysis

High-performance thin layer chromatography (HPTLC) was performed using the Automatic Development Chamber 2 (ADC 2, CAMAG®, Muttenz, Switzerland), with HPTLC silica gel 60 F254 plates (20 × 10 cm, particle size 5–6 μm, glass support with fluorescent indicator, Merck KGaA, Darmstadt, Germany). The mobile phase was prepared as a mixture of purified water, methanol, acetic acid and dichloromethane in the ratio 2:3:8:15 (v/v). The reference solution was prepared as a mixture of caffeic acid and chlorogenic acid in methanol (ANSM, 2010).

Two microliters of the VAMT and reference solution were applied using an Automatic TLC Sampler 4 (ATS 4, CAMAG®). The dispersion distance was set to 70 mm, with a drying time of 5 min. Each sample was applied as 10 mm bands, with an 8 mm distance from the lower edge of the plate. After the run, the samples were revealed with diphenylboryloxyethylamine (NP) and polyethylene glycol (PEG), which was applied with an automated spraying (CAMAG® Derivatizer). The plate was subsequently heated at 110 °C on a TLC Plate Heater 3 (CAMAG®) for 6 min. Visual inspection and photography of the plate were performed at wavelengths of 254 nm, 366 nm, and under white light. The samples were characterized by their respective retention factors (Rf) in relation to the reference solutions (ANSM, 2010).

### 2.3 Quantification of total flavonoids

The total flavonoid content of each VAMT was determined spectrophotometrically in the UV region (360 nm), using a rutin standard curve. To prepare the curve, 25 mg of rutin was dissolved in 50 mL of a mixture of ethanol 96° GL and 0.02 M acetic acid (99:1). The following rutin concentration range was used: 5.0, 10.0, 15.0, 20.0, 25.0, and 30.0 μg/mL 500 μL of all winter and summer VAMT were centrifuged in three different Eppendorf tubes for 5 min at 14,000 rpm. Afterwards, the supernatants were removed, and the centrifugation was repeated. At the end of the process, 200 µL of each VAMT were transferred to a 10 mL volumetric flask, and the volume was adjusted with the same solvent used in all solution preparations. The experiment was performed in triplicate, and the readings were taken with a spectrophotometer (Genesys 10S UV-VIS - Thermo Scientific) set to 360 nm (Holandino et al., 2020).

### 2.4 Cell culture

The porcine kidney proximal tubule cell line (LLC-PK1) was grown in Dulbecco’s Modified Eagle Medium (DMEM) Low Glucose with 10% inactivated foetal bovine serum (FBS), penicillin at 100 IU/mL, streptomycin at 100 μg/mL and maintained in a cell incubator with 5% CO_2_ at 37 °C.

### 2.5 Evaluation of cytotoxicity by MTT assay

For the MTT assay, cells were seeded into a 96-well plate, with 100 µL of cell suspension containing 2 × 10^4^ cells per well. After 24 h, 100 µL of each VAMT solution, prepared at concentrations ranging from 0.5% to 2.5% v/v, were added to the respective wells. The control solutions included a 60% v/v ethanol solution (matching the hydroalcoholic content of the extracts) and a negative control (untreated cells) (Holandino et al., 2020; Melo et al., 2022). After 24 h of incubation in a humidified atmosphere with 5% CO_2_ at 37 °C in the dark, the test solutions were replaced by low-glucose DMEM medium containing MTT solution at 5 mg/mL. The plates were then incubated under the same conditions for 3 h. Following incubation, the plates were centrifuged at 1,218 g for 8 min. The resulting formazan crystals were dissolved in 200 µL of dimethyl sulfoxide (DMSO), and absorbance was measured at 490 nm using a multiwell plate reader (Thermo Plate, TP-Reader). Cell viability was calculated relative to the control groups (untreated and ethanol-treated cells) based on mean values from five independent experiments performed in quadruplicate. The percentage of viable cells was analyzed using GraphPad Prism 5.

### 2.6 Reactive oxygen species assay (ROS)

Cells were seeded into a 96-well plate at a density of 2 × 10^4^ cells per well. After 24 h of incubation at 37 °C in a humidified atmosphere containing 5% CO_2_, VAMT extracts from *M. domestica*, *P. sylvestris* and *A. alba* host trees were added at a concentration of 1.5% v/v. The following control groups were included: negative control (low glucose DMEM with 10% FBS), solvent control (1.5% hydroethanolic solution at 60% v/v), and a positive control (600 µM of hydrogen peroxide incubated only for 30 min). After the designated incubation period, cell supernatants were removed, and 30 µM of 2′,7′-Dichlorofluorescein diacetate (H2DCFDA) was added to each well. Following an additional 30-min reaction period, the wells were washed twice with 100 µL of phosphate-buffered saline (PBS). Finally, 100 µL of PBS were added to each well, and fluorescence was measured using a spectrofluorometer (Spectra Max M5) at excitation/emission wavelengths of 495 nm/530 nm (Bonaterra et al., 2022). Six independent experiments were done in triplicate.

### 2.7 Protein determination

Ten microliters of each VAMT sample were used for protein assays (in triplicate). The Folin phenol method was performed with addition of 5% of sodium dodecyl sulphate (Lowry et al., 1951). A standard bovine serum albumin (BSA) curve was prepared using the following BSA concentrations: 10, 20, 30, 40, 50 μg/mL (Cabral et al., 2007).

### 2.8 Determination of the Na^+^/K^+^ ATPase activity

Cells were cultured in a 6-well plate at a density of 4 × 10^5^ cells per well, incubated in a humidified atmosphere with 5% CO_2_ at 37 °C for 24 h. The confluent monolayer was then treated with winter and summer VAMT extracts from *M. domestica*, *P. sylvestris* and *A. alba* host trees at a concentration of 1.5% v/v for 30 min and 24 h. Control groups included cells with culture medium, cells treated with 60% v/v hydroethanolic solution, and cells treated with the Na^+^/K^+^ ATPase inhibitor (ouabain), at 10 mM. The methodology followed previously published protocols by our group (Sampaio et al., 2018; Taveira da Silva et al., 2019), with at least six independent experiments conducted in triplicate. After treatment, enzyme activity was assessed by measuring absorbance at 660 nm using a spectrophotometer (Genesys 10S UV-VIS - Thermo Scientific) as described by Fiske and Subbarow (1925).

### 2.9 Western blotting

Cells were cultured in a 6-well plate at a density of 4 × 10^5^ cells per well, incubated in a humidified atmosphere with 5% CO_2_ at 37 °C for 24 h, ensuring approximately 80% confluence. After this incubation period, winter, and summer extracts from *M. domestica*, *P. sylvestris*, and *A. alba* were added at a concentration of 1.5% v/v (tested samples), along with control groups consisting of cellular medium and at 60% v/v hydroethanolic solution. An additional incubation was conducted for 24 h. On the third day of the experiment, cells were lysed using a buffer containing 10 mM EDTA, 50 mM HEPES-Tris, 1 M sucrose, trypsin inhibitor, and ultrapure water. A cell scraper was used to collect remaining cells, which were then combined with those lysed by the buffer. Total cellular protein was quantified using the Lowry method (Lowry et al., 1951), and 15 µg from each experimental group were separated by sodium dodecyl sulphate-polyacrylamide gel electrophoresis (SDS-PAGE) at 10%, ensuring high-resolution separation of the complex protein mixture. At the end of the SDS-PAGE process, the cellular proteins were transferred to a nitrocellulose membrane. The membranes were incubated with a mouse anti alpha 1 Na^+^/K^+^ ATPase monoclonal antibody (1:1000) and a mouse anti-GAPDH monoclonal antibody (1:1000) (Taveira da Silva et al., 2019; Silva, 2013). After washing with TTBS (TBS [50 mM Tris-HCl at pH 7.4, 150 mM NaCl] containing 0.1% Tween 20), the membranes were incubated with SuperSigmal West Pico Plus (Thermo Scientific) and specific proteins were visualized with Image Quant LAS 4000 (General Electric). The intensity of specific bands was analyzed by ImageQuant and measured using ImageJ.

### 2.10 Effects on the peripheral vascular resistance of hypertensive rats

#### 2.10.1 Animals

Male spontaneously hypertensive rats (SHR), weighing 280–310 g and obtained from the central animal facility of the Federal University of Grande Dourados (UFGD, Brazil), were used in this study. The animals were provided with food and water *ad libitum* under controlled conditions: ambient temperature (22 °C ± 2 °C), humidity (50% ± 10%), and 12 h light/dark cycle (lights on at 07:00 a.m.). All experimental procedures were conducted in accordance with the Guidelines for the Care and Use of Laboratory Animals adopted by the U.S. National Institutes of Health. Additionally, the study was approved by the Institutional Ethics Committee of UFGD (authorization number 24009).

#### 2.10.2 Investigation of the vasodilatory effects of winter and summer VAMT from *A. alba*, *Malus domestica*, and *P. sylvestris* on MVBs from SHR

Initially, SHR were anesthetized intramuscularly with ketamine (100 mg/kg) and xylazine (20 mg/kg). The MVBs were then isolated and prepared as previously described (McGregor, 1965; Tolouei et al., 2019a; Tolouei et al., 2019b). The MVBs were excised and mounted in a water-jacketed organ bath maintained at 37 °C. The preparations were perfused at a constant flow rate of 4 mL/min with physiological saline solution (PSS) aerated with a carbogen mixture (95% O_2_/5% CO_2_). The PSS composition was (in mM): NaCl 119, KCl 4.7, CaCl_2_ 2.4, MgSO_4_ 1.2, NaHCO_3_ 25.0, KH_2_PO_4_ 1.2, dextrose 11.1, and EDTA 0.03 (pH 7.4). Following a 30–45-min equilibration period, tissue viability was assessed via a bolus injection of KCl (120 mmol). Changes in perfusion pressure (mmHg) were monitored using a pressure transducer coupled to a PowerLab® data acquisition system running Chart v8.1 software (AD Instruments, Castle Hill, Australia). Then, endothelium-intact MVBs were continuously perfused with PSS containing phenylephrine (Phe 3 µM). Once a stable contraction plateau was reached, endothelial functional integrity was verified by recording the vasodilatory response to a bolus injection of acetylcholine (ACh, 1 nmol; positive control). Additionally, a bolus of PSS (negative control) was administered to all pre-contracted preparations to confirm the absence of vehicle-induced vasodilation. Subsequently, dose-response curves were generated by administering bolus injections (0.003, 0.01, 0.03, 0.1, 0.3, and 1 mg) of freeze-dried winter and summer VAMT from *A. alba*, *M. domestica*, and *P. sylvestris*. Doses were administered at 3-min intervals, and perfusion pressure changes were recorded. Six preparations per group were used in this assay.

#### 2.10.3 Investigation of the molecular mechanisms underlying the vasodilatory effects induced by winter VAMT from *P. sylvestris* (VAMT PW)

To investigate the molecular mechanisms underlying the vasodilatory activity, experiments were conducted exclusively using the VAMT PW. Initially, a stable contractile tone was established by continuous perfusion with PSS containing 3 µM of phenylephrine (Phe), followed by the generation of a dose-response curve for VAMT PW (0.01, 0.03, and 0.1 mg). Subsequently, separated preparations were perfused with PSS containing Phe (3 µM) in the presence of specific inhibitors or channel blockers: L-NAME (100 μM, a non-selective nitric oxide synthase [NOS] inhibitor), indomethacin (1 μM, a non-selective cyclooxygenase inhibitor), KCl (40 mM), tetraethylammonium (10 mM, a non-specific potassium channel blocker), glibenclamide (10 μM, an ATP-sensitive potassium (K (ATP)) channel blocker), 4-aminopyridine (4-AP) (10 μM, a voltage-gated potassium (Kv) channel blocker), iberiotoxin (10 nM, a large-conductance calcium-activated potassium channel, BKCa), charybdotoxin (10 nM, an intermediate- and large-conductance calcium-activated potassium channel, IKCa and BKCa), or apamin (10 nM, a small-conductance calcium-activated potassium channel, SKCa). After a 15-min equilibration period with the respective inhibitor, VAMT PW (0.01, 0.03, and 0.1 mg) was re-administered. The vasodilatory effect of VAMT PW under these conditions was compared to control responses obtained in preparations perfused solely with the vehicle (PSS). Six preparations per group were used in this assay.

### 2.11 Statistical analysis

The data are expressed as the mean ± standard error of the mean (SEM) for independent experiments in the *in vitro* assays, or for six preparations per group in the *in vivo* study. Statistical comparisons were performed using one-way analysis of variance (ANOVA), unpaired t-test, and Student’s t-test considering the experimental groups. The Dunnet, Tukey and Bonferroni post-tests were used to identify differences among groups. A p-value <0.05 was considered statistically significant. All statistical analyses and graphical representations were generated using GraphPad Prism 10 for macOS (GraphPad Software, Boston, MA, United States).

## 3 Results

### 3.1 Cytotoxicity by MTT

**FIGURE 1:**
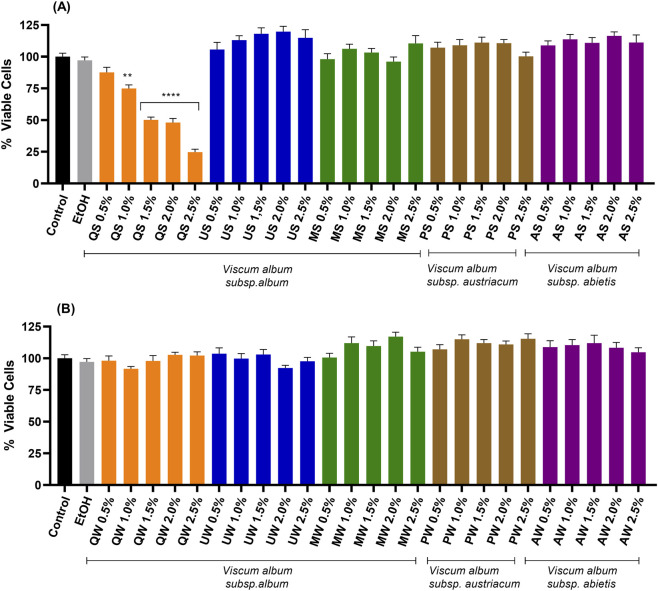
MTT cytotoxic assay induced by *V. album* mother tinctures (VAMTs) and respective controls in LLC-PK1 cells. Panel **(A)**: effects of summer VAMTs; Panel **(B)**: effects of winter VAMTs. Control (cells incubated in culture medium), EtOH (ethanol vehicle control). VAMTs were prepared using *V. album* harvested in summer (S) and winter (W) from the following host trees: *Quercus petraea* (QS; QW), *Ulmus carpinifolia* (US; UW), *Malus domestica* (MS; MW), *Pinus sylvestris* (PS; PW) and *Abies alba* (AS; AW). Statistical analysis was performed using One-Way ANOVA with Tukey as post-test. The mean ± standard error was obtained from six independent experiments performed in quintuplicate. **(p < 0.01); ***(p < 0.001) compared to untreated cells (control; black bars).

Considering all VAMT tested, only mistletoe sample from the *Q*. *petraea* host tree, harvested in summer, showed dose-dependent cytotoxicity against LLC-PK1 cells (Figure 1A), with the highest cytotoxic effect observed after 24 h of incubation with QS 2.5% v/v (p < 0.001). Therefore, *V. album* harvested in summer from *Q*. *petraea* was excluded from further investigation. Conversely, the other VAMT prepared with summer (Figure 1A) and winter harvests (Figure 1B) induced no statistically significant differences among groups.

According to the results presented in Figure 1, VAMT derived from the following host trees - *M. domestica*, *P*. *sylvestris*, and *A*. *alba* (both winter and summer samples)—were selected for the other experimental investigations. Only one host tree of each *V. album* subspecies was selected for this study, based on previous results from our group (Melo et al., 2022).

### 3.2 Quantification of total flavonoids

Table 1 shows the results of total flavonoids content as rutin equivalents of VAMT from different host trees (*A*. *alba, M*. *domestica*, *P*. *sylvestris*) from winter (AW, PW, MW) and summer (AS, PS, MS). The total flavonoids content of summer samples was higher (*p* < 0.05) than winter samples, excepted for VAMT from *M*. *domestica*. Among all the VAMTs analyzed, the summer extract of *V. album* from *A. alba* and *P*. *sylvestris* exhibited the highest total flavonoid content (Table 1), with no significant statistical difference between these two samples. These data demonstrated a relationship between the host tree and the flavonoid content, highlighting the importance of traceability regarding harvest patterns for the use of *V. album* mother tinctures.

**TABLE 1:** Total flavonoid content in rutin equivalents.

Mother tinctures	Mean ± standard deviation (µg/mL)
AS	1.67 ± 0.06^a,b,c,d^
AW	1.02 ± 0.31^a^
PS	1.43 ± 0.19^e,f^
PW	0.90 ± 0.04^b,e^
MS	1.08 ± 0.08^c^
MW	1.00 ± 0.03^d,f^

Legend: Mother tinctures samples were prepared with *V. album* harvested from different host trees and season, as follow: AS (*Abies alba* - summer), AW (*Abies alba* - winter), PS (*Pinus sylvestris* - summer), PW (*Pinus sylvestris* - winter), MS (*Malus domestica* - summer), MW (*Malus domestica* - winter). *p < 0.05 (e,f); **p < 0.005 (a,c,d); ***p < 0.0005 (b). Equal letters represent the comparison among groups.

### 3.3 HPTLC

**FIGURE 2:**
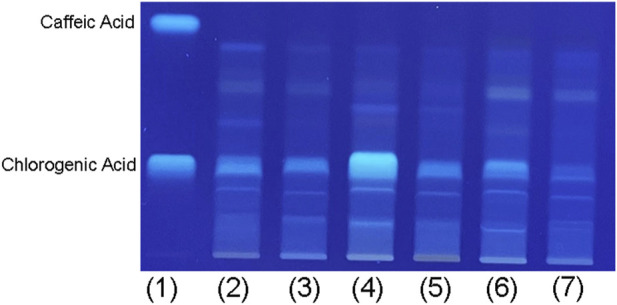
High-performance thin layer chromatography (HPTLC) of *V*. *album* mother tinctures (VAMTs), visualization at 366 nm. Samples: (1) Standard mixture of caffeic acid and chlorogenic acid, (2) *V. album* from *Malus domestica* - summer, (3) *V. album* from *Malus domestica* - winter, (4) *V. album* from *Abies alba* - summer, (5) *V. album* from *Abies alba* - winter, (6) *V. album* from *Pinus sylvestris* - summer, (7) *V. album* from *Pinus sylvestris -* winter.

The HPTLC analysis (Figure 2) revealed bluish bands under UV light at 366 nm, which is characteristic of phenolic acids (Wagner and Bladt, 2001). The samples confirmed the presence of chlorogenic acid with Rf 0.40, approximately, indicated by a bluish colour band that matched the chemical standard used for comparison. However, caffeic acid (Rf 0.90) was not detected in the VAMT samples. The summer *V. album* from *A*. *alba* sample showed a more intense size and colour band of chlorogenic acid (Figure 2, line 4). The chemical profile identified by HPTLC analysis of VAMT was in accordance with the French Pharmacopoeia (ANSM, 2010), which was used for quality control of the samples prior to further *in vitro* and *in vivo* studies.

### 3.4 Oxygen-reactive species (ROS)

**FIGURE 3:**
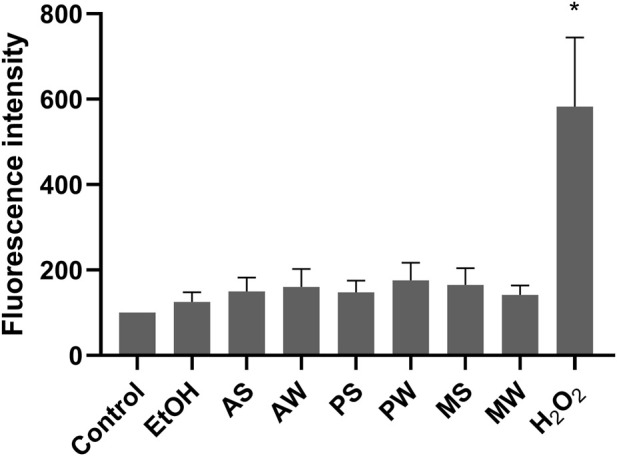
ROS generated by LLC-PK1 after 24 hours of incubation with VAMTs and respective controls. Control (cells incubated in culture medium); EtOH (cells incubated in ethanol vehicle control); H_2_O_2_ (cells incubated in hydrogen peroxide solution - positive control). VAMTs were prepared using *V. album* harvested in summer (S) and winter (W) from the following host trees: *Abies alba* (AS; AW), *Pinus sylvestris* (PS; PW) and *Malus domestica* (MS; MW). Statistical analysis was performed using One-Way ANOVA with Dunnett as post-test. The mean ± standard error was obtained from five independent experiments performed in triplicate. ∗p < 0.05 compared to the positive control.

Although the antioxidant activity of *V. album* is well documented (Olas, 2024; Stefanucci et al., 2020), the determination of reactive oxygen species (ROS) was performed to evaluate the behaviour of VAMT in relation to the cell type studied (LLC-PK1). No increase in ROS generation was detected in the cell supernatant, except in the positive control group, hydrogen peroxide, incubation (Figure 3). Our results indicate that VAMT does not induce oxidative stress under the experimental conditions tested.

### 3.5 Evaluation of Na^+^/K^+^ ATPase activity

**FIGURE 4:**
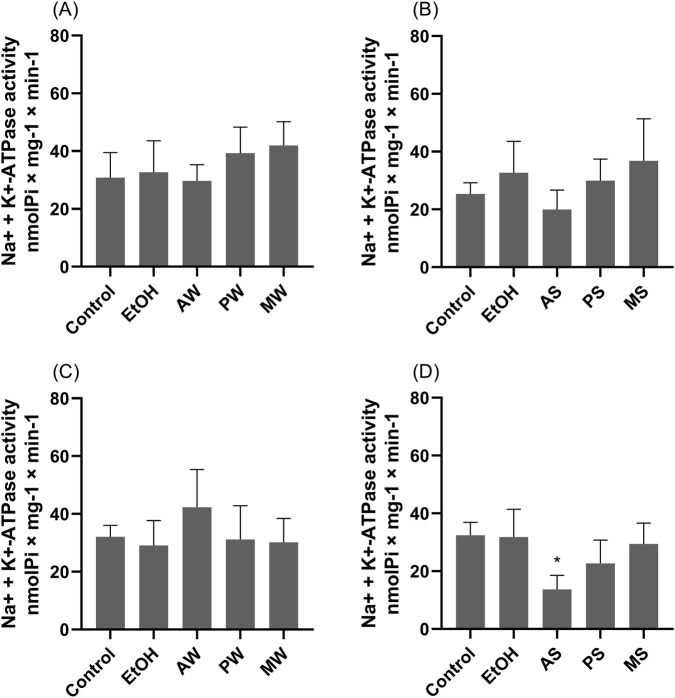
Na^+^/K^+^ ATPase activity of LLC-PK1 cells measured after 30 min **(A, B)** and 24 hours **(C, D)** of VAMTs incubation. Control (cells incubated in culture medium); EtOH (cells incubated in ethanol vehicle control). VAMTs were prepared using *V. album* harvested in summer (S) and winter (W) from the following host trees: *Abies alba* (AS; AW), *Pinus sylvestris* (PS; PW) and *Malus domestica* (MS; MW). Statistical analysis was performed using One-Way ANOVA with Tukey as post-test. The mean ± standard error was obtained from six independent experiments performed in triplicate. ∗p < 0.05 compared to the control.

Na^+^/K^+^ ATPase activity in LLC-PK1 cells was unaffected by 30-min treatment with 1.5% v/v VAMT from *M*. *domestica*, *P*. *sylvestris*, and *A*. *alba* (Figures 4A,B). However, after 24 h, the summer VAMT of *A*. *alba* significantly reduced enzyme activity by 42% compared to the negative control (cells in culture medium; p < 0.05) (Figure 4D). These results highlight incubation time as a key factor in assessing Na^+^/K^+^ ATPase activity, with longer exposure revealing a notable enzymatic inhibition by the summer VAMT from *A*. *alba*.

### 3.6 Western blotting

**FIGURE 5:**
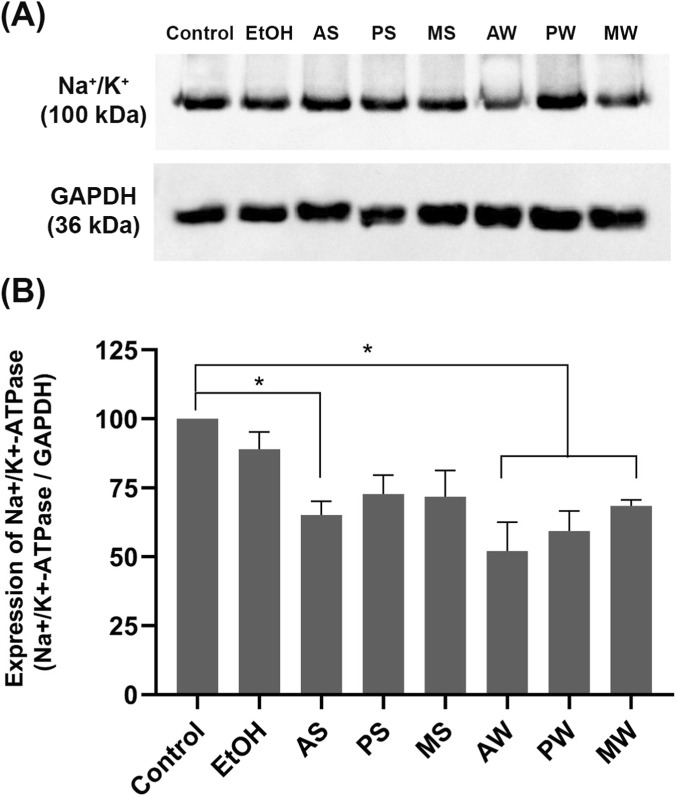
LLC-PK1 expression of Na^+^/K^+^ ATPase evaluated by western blotting. (A) Upper panel: immunodetection of the pump, performed according to the described methodology; Lower panel: immunodetection of GAPDH, performed according to the described methodology. **(B)** quantification of pump expression: the graph shows the ratio of Na^+^/K^+^ ATPase densitometry to GAPDH densitometry for each experimental group. Control (cells incubated in culture medium); EtOH (cells incubated in ethanol vehicle control). VAMTs were prepared using *V. album* harvested in summer (S) and winter (W) from the following host trees: *Abies alba* (AS; AW), *Pinus sylvestris* (PS; PW) and *Malus domestica* (MS; MW). Statistical analysis was performed using One-Way ANOVA with Dunnett as post-test. The mean ± standard error was obtained from three independent experiments. ∗p < 0.05 compared to the control.

The *in vitro* results indicate that the treatment with VAMT for 24 h induced a decrease in the expression of Na^+^/K^+^ ATPase. Figures 5A,B show a 32%–48% reduction in the expression of this enzyme compared to the control after 24 h of incubation with AS, AW, MW and PW. Ethanol treatment did not cause a statistically significant difference relative to the control, excluding effects from the extraction solvent. Notably, the summer VAMT from *A*. *alba* induced the greatest reduction in Na^+^/K^+^ ATPase activity (Figure 4D) and significantly decreased pump expression by 35% compared to the control (p < 0.05). Conversely, the winter VAMT from the same host tree reduced pump expression by 48%. Regarding the other host trees, only the winter extracts promoted a decrease in Na^+^/K^+^ ATPase expression. GAPDH was used as an internal control for normalization.

It is worth highlighting that the winter samples, and especially those of *V. album* from hosts *A*. *alba* and *P*. *sylvestris*, were the ones responsible for the strongest effects detected in LLC-PK1 cells. This is the first study to examine the impact of *V. album* mother tinctures on this essential pump involved in Na + reabsorption.

### 3.7 Vasodilator effects of winter and summer VAMTs from *A. alba*, *Malus domestica*, and *P. sylvestris*

**FIGURE 6:**
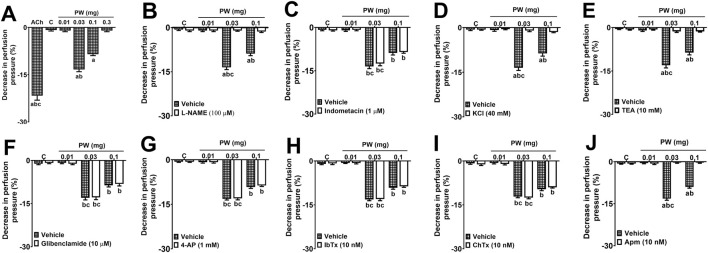
Role of the NO/cGMP pathway and SKCa channel activation in the vasodilator effects of VAMT from *Pinus sylvestris* winter (PW) **(A)** Dose-response vasodilator effects of PW on mesenteric vascular beds (MVB) pre-contracted with phenylephrine (Phe; 3 µM) **(B–J)** Effects of VAMT-PS-Winter (0.01, 0.03, and 0.1 mg/kg) on MVB continuously perfused with Phe (3 µM) plus L-NAME **(B)**, indomethacin **(C)**, 40 mM KCl **(D)**, tetraethylammonium (TEA, **E**), glibenclamide **(F)**, 4-aminopyridine (4-AP, **G**), iberiotoxin (IbTx, **H**), charybdotoxin (ChTx, **I**), or apamin (Apm, **J**). Results are shown as the mean ± S.E.M. of six preparations per group. Comparisons between the different doses of VAMT-PS-Winter were conducted using one-way ANOVA followed by the Bonferroni test. The difference between the inhibitor/antagonist groups and the vehicle-only group, at each dose, was determined using Student’s t-test. In graph A, **(A)** p < 0.05 compared with the negative control (C, nutritive solution), VAMT-PS-Winter 0.01, or 0.3 mg; **(B)** p < 0.05 compared with VAMT-PS-Winter 0.1 mg; and **(C)** p < 0.05 compared with VAMT-PS-Winter 0.03 mg. In graphs **(B–J)**, **(A)** p < 0.05 compared to the respective vehicle group; **(B)**, p < 0.05 compared with the negative control (C, nutritive solution) or VAMT-PS-Winter 0.01 mg; and **(C)**, p < 0.05 compared with VAMT-PS-Winter 0.1 mg. All experiments were performed on endothelium-intact preparations.

Freeze-dried VAMT from *A. alba* and *M. domestica* (summer and winter), as well as summer *P. sylvestris* (0.003–1 mg), did not elicit significant vasodilatory responses compared to the vehicle control (PSS; *data not shown*). In contrast, Figure 6A shows the dose-response effects of bolus injections (0.01–0.3 mg) of freeze-dried VAMT from winter *P. sylvestris* (PW). In preparations with functional endothelium, doses of 0.03 and 0.1 mg reduced perfusion pressure by approximately 12 ± 2.1 and 9 ± 2.3 mmHg, respectively (Figure 6A; p < 0.05).

### 3.8 Molecular mechanisms involved with the vasodilatory effects of VAMT from *P. sylvestris* (VAMT PW)

Since only the VAMT PW exhibited significant vasodilator activity in the MVBs, the investigation into the underlying molecular mechanisms was restricted to this specific preparation. Inhibition of NOS with L-NAME completely abolished the vasodilation induced by all doses of VAMT PW (Figure 6B). Similarly, perfusion with a physiological solution containing high KCl (40 mM) fully eliminated the vasodilatory effect (Figure 6D). Furthermore, pre-treatment with tetraethylammonium (TEA), a non-selective potassium channel blocker, and apamin, a blocker of SKCa channels, fully inhibited the vasodilatory responses induced by VAMT PW (Figures 6E,J). In contrast, indomethacin, 4-aminopyridine (4-AP), glibenclamide, iberiotoxin, and charybdotoxin did not significantly alter the vasodilation promoted by VAMT-PS-Winter (Figures 6C,F–I).

## 4 Discussion

In the first phase of this study, we investigated the cytotoxic potential of VAMT obtained from different host trees. Cytotoxicity screening is pivotal not only to ensure consumer safety but also to define the therapeutic window, distinguishing beneficial low doses from potentially toxic high doses. Our findings serve as a preliminary safety validation, supporting the transition from *in vitro* screening to subsequent *in vivo* assays (Niles et al., 2008). The observed cytotoxic effect in the VAMT from *Quercus petrea* summer (Figure 1A) may be related to the higher level of viscotoxins present in this sample (3.38 mg/g fresh plant; Suplementary Material). This finding is consistent with previous data from our group, which showed that another summer VAMT from *Quercus spp* exhibited high levels of viscotoxins (3.08 mg/g fresh plant) and induced necrotic effects in a tumour cell culture assay (Holandino et al., 2020). These results highlight the impact of seasonality on the cytotoxic potential of VAMT, with summer preparations being more than winter ones.

Given that VAMT derived from *M. domestica*, *A. alba* and *P. sylvestris* host trees exhibited no significant cytotoxicity, we proceeded to chemical analysis of these preparations. Notably, the analysis revealed a high content of flavonoids and phenolic acids, predominantly chlorogenic acid. These compounds are well-recognized modulators of physiological processes, known for their anti-inflammatory, antioxidant, diuretic, antihypertensive, and vasodilatory properties (Kumar and Goel, 2019; Stachelska et al., 2025). Among all analyzed samples, the VAMT from *A. alba* and *P. sylvestris* exhibited the highest total flavonoids content (p < 0.05) (Table 1). These findings suggest a clear influence of the host tree on the flavonoid and phenolic acids content, highlighting the importance of harvest traceability in the preparation of VAMTs (Holandino et al., 2020; Melo et al., 2018). The comparison with previous findings reported by Holandino et al. (2020) provides valuable insights into the influence of season and year of harvests on the phytochemical composition of *V. album*. In that study, which analyzed summer mother tinctures obtained in 2016, *V. album* growing on *Quercus robur* exhibited the highest total flavonoid content, followed by samples from *M. domestica*, *A. alba*, *U. carpinifolia*, and *P. sylvestris*. In the present work, the differences in flavonoid concentration relative to those earlier results may be largely explained by temporal variation in harvests—specifically, the inclusion of material collected in summer 2017 and winter 2018. Seasonal fluctuations in temperature, light exposure, and host–parasite interactions are known to modulate *V. album* secondary metabolism, leading to variations in its bioactive compound profile (Escher et al., 2004; Soursouri et al., 2023).

Consistent with the antioxidant potential attributed to the flavonoids and phenolic acids identified in the VAMTs, our study also revealed that the treatment did not increase intracellular ROS levels (Figure 3). This finding aligns with reports describing similar antioxidant behaviours in plant-derived extracts. For instance, Lee et al. (2019) reported that treatment of LLC-PK1 cells with the compound 3-dehydroxyceanothetric acid 2-methyl ester from *Ziziphus jujuba* not only failed to elevate ROS levels, but significantly reduced them in cells exposed to cisplatin, suggesting a protective antioxidant effect. Similarly, Hu et al. (2023) demonstrated that methanolic extracts of *Peucedanum praeruptorum* did not induce ROS accumulation in LLC-PK1 cells; instead, they caused a marked decrease in ROS levels, supporting their ROS-scavenging potential. It is well established that increased generation of ROS contributes to the genesis of arterial hypertension and endothelial dysfunction. The superoxide anion reacts with nitric oxide (NO), a vasodilator, forming peroxynitrite, a potent oxidant. Furthermore, oxidative stress leads to a reduction in the cofactor tetrahydrobiopterin, resulting in the uncoupling of endothelial nitric oxide synthase (eNOS), which further increases peroxynitrite production (Piacenza et al., 2022). Collectively, these effects contribute directly to endothelial dysfunction and the establishment of hypertension.

The Na^+^/K^+^ ATPase is recognized for its dual role in long-term blood pressure control, a function of particular significance given the global burden of hypertension. At the renal level, a reduction in the expression or activity of this enzyme decreases tubular Na + reabsorption, promoting natriuresis and potentially lowering blood pressure under specific conditions, as it involves tubular sodium reabsorption and extracellular fluid volume (McDougall and Yates, 1998). Additionally, this enzyme acts not only as an ion pump but also as a signalling molecule crucial for vascular tone regulation, modulating smooth muscle Ca^2+^ sensitivity via Src kinase signalling (Staehr et al., 2023).

Based on this essential physiological control, the present work investigated the modulation of Na^+^/K^+^ ATPase using renal proximal tubular epithelial cells. Our *in vitro* results showed that, among all tested *V. album* formulations, the VAMT derived from *A. alba* in the summer and all VAMTs harvested in the winter significantly reduced Na^+^/K^+^ ATPase activity and expression, respectively (Figures 4, 5). This downregulation induced by specific VAMT preparations is a significant finding and highlights, for the first time, that both season and host tree are critical determinants of this modulation.

Similar studies have already been conducted with other plant species, showing changes in the activity and expression of Na^+^/K^+^ ATPase triggered by these natural products. For instance, Jaber et al. (2013) reported a significant decrease in this enzyme’s activity in diabetic rat models following the administration of an aqueous extract of banana infructescence stalks. Conversely, Müller et al. (2015) showed an increase in Na^+^/K^+^ ATPase activity in the cortex and hippocampus of mice treated with *Valeriana glechomifolia* Meyer, a plant noted for its anti-inflammatory and antidepressant properties. Additionally, Qu et al. (2019) reported that a mixture of green and black tea led to a reduction in this pump’s activity in a diabetic mouse model. These findings indicate that the effects on Na^+^/K^+^ ATPase activity can vary significantly depending on the specific plant extract employed. Suzuki et al. (2006) demonstrated that, regardless of the exposure model or extract tested, Na^+^/K^+^ ATPase expression tended to increase following treatment with various plant-derived preparations. Moreover, *Alpinia oxyphylla* extract exhibited a protective effect on protein expression in a rat model of acute renal failure induced by ischemia and reperfusion (Kim et al., 2013). Given the established evidence linking ROS and Na^+^/K^+^ ATPase activity to renal function and arteriolar tone regulation—a critical determinant of peripheral resistance and blood pressure—we investigated the effects of VAMTs on arteriolar resistance. Hypertensive phenotypes are frequently characterized by elevated oxidative stress, altered Na^+^/K^+^ ATPase expression, and sustained increases in arteriolar tone. These alterations are partially driven by a significantly impaired NO-mediated vasodilatory response, leading to endothelial dysfunction. To elucidate the molecular mechanisms underlying the modulation of arteriolar tone by VAMTs, we utilized MVBs isolated from SHR. Our findings demonstrate that VAMT-PS-Winter induces sustained vasodilation in resistance arteries. The data indicate an upregulation of the NO/soluble guanylate cyclase (sGC)/increasing cyclic guanosine monophosphate (cGMP) pathway, culminating in the opening of SKCa channels and subsequent vasodilation. Current literature suggests that flavonoids and phenolic acids enhance intracellular calcium concentrations within the vascular endothelium, thereby activating eNOS and elevating NO levels in vascular smooth muscle (Kant, Sellke and Feng, 2022). Subsequently, NO activates sGC, increasing cGMP levels and activating protein kinase G (PKG) (Gambaryan, 2022). PKG promotes vasodilation by opening calcium-activated potassium channels and stimulating myosin light chain phosphatase (MLCP), which decreases myosin light chain phosphorylation (Ran et al., 2024). We hypothesize that the observed vasodilatory effect is largely attributable to the reduction of oxidative stress by secondary metabolites, such as phenolic acids and flavonoids, present in VAMT-PS-Winter, alongside the direct modulation of Na^+^/K^+^ ATPase expression. Our results suggest that VAMTs induce a complex, multi-target pharmacological profile, in which moderate modulation of Na^+^/K^+^ ATPase expression occurs alongside dominant endothelium-dependent vasodilatory mechanisms. To confirm our hypothesis, future investigations are warranted to evaluate the antihypertensive and cardioprotective potential of VAMT-PS-Winter in *in vivo* experimental hypertension models, verifying whether the mechanisms identified in our *ex vivo* assays translate to systemic efficacy.

Although VAMTs induce a complex, multi-target pharmacological profile—characterized by moderate modulation of Na^+^/K^+^ ATPase expression alongside dominant endothelium-dependent vasodilatory mechanisms—a key aspect remains to be elucidated. In resistance vessels, such as MVBs, the primary NO signaling pathway is cGMP-dependent, wherein PKG reduces intracellular calcium levels to promote vasodilation (Kemp-Harper and Schmidt, 2009; Gambaryan, 2022). Conversely, while the cGMP pathway may be present in LLC-PK1 cells, NO signaling in this cell line is frequently associated with cGMP-independent pathways, such as S-nitrosylation of proteins and activation of the PI3K/Akt signaling cascade (Khalil et al., 2022). Furthermore, NO can modulate ion transporters (including Na^+^/K^+^ ATPase) and influence the stabilization of transcription factors such as HIF-1α (Hypoxia-Inducible Factor 1-alpha), facilitating adaptation to oxidative stress or inflammation (Zhou et al., 2003). Consequently, future studies should be designed to investigate the crosstalk between NO signaling pathways in LLC-PK1 cells and the vessels that comprise peripheral vascular resistance.

Another aspect requiring clarification concerns the dose-response profile observed in this study. Vasodilatory activity was restricted to specific doses (0.03 and 0.1 mg), with the effect being attenuated at higher concentrations. This phenomenon, characterized as a non-monotonic or inverted U-shaped dose-response curve, is frequently observed in plant extracts. In such complex matrices, various bioactive molecules may act through distinct pharmacological pathways (Nguelefack-Mbuyo et al., 2023). It is plausible that at 0.03 and 0.1 mg, vasodilatory compounds predominated, leading to a significant reduction in peripheral resistance. Conversely, at higher doses, the recruitment of molecules with antagonistic or hypertensive properties may have counteracted the initial vasodilation. Consequently, a significant limitation of this study is the failure to identify the specific secondary metabolites responsible for the vasodilatory effects of VAMT-PS-Winter. Nevertheless, previous phytochemical profiling performed by our group identified phenolic acids and flavonoids as the major chemical constituents in other VAMT samples (Melo et al., 2018; Holandino et al., 2020). These classes, particularly flavonoids, are well-documented for their cardioprotective and vasorelaxant properties (Cao et al., 2021; Ciumărnean et al., 2020). Future research should focus on the bio-guided fractionation of VAMT-PS-Winter to isolate active compounds and assess whether this *in vitro* vasodilation translates into systemic hemodynamic improvements in chronic hypertensive models.

## 5 Conclusion

The present study demonstrated that *V. album* mother tinctures sourced from *A. alba*, *M. domestica*, and *P. sylvestris* exhibit no cytotoxicity toward renal cells and do not induce *in vitro* oxidative stress. Regarding molecular targets, distinct pharmacological profiles were identified: while VAMT from *Abies alba*, harvested in summer, diminished activity of Na^+^/K^+^ ATPase, all VAMT, harvested in winter, downregulated the enzyme expression. Besides, only VAMT from *P. sylvestris*, harvested in winter, elicited significant vasodilation in mesenteric resistance arteries from hypertensive rats. This vasorelaxant effect is likely mediated via the NO/sGC/cGMP signaling pathway and subsequent activation of SKCa channels. Collectively, our findings highlight the critical influence of the host tree and harvest season on the biological activity of *V. album*, positioning VAMT from *P. sylvestris* as a promising candidate for further pharmacological development in cardiovascular research.

## Data Availability

The raw data supporting the conclusions of this article will be made available by the authors, without undue reservation.
