# Supplementary material for: Viscum album L. mother tinctures modulate Na+/K+ ATPase activity and expression, and promote endothelium-dependent vasodilation via SK channel and nitric oxide signalling
Source: Front Physiol. 2026 Feb 2;16:1736143. doi: 10.3389/fphys.2025.1736143 (PMC12907139; doi:10.3389/fphys.2025.1736143)
Supplement: Supplementary file 1 [file Table1.pdf]

Supplementary material: Duarte et al., 2025

Table S1: Data on the production of VAMTs.

Season	Host Tree	Fresh material (g)	Dry residue (%)	Total solid residue (g)	Total TM volume mL (10x dry residue)	Water in plant	Alcohol to add mL	*Viscotoxin/ Fresh material (mg/g)	SD
Winter	*Pinus silvestris*	21.4113	37.84	8.10	81.01	13.31	67.70	0.20	0.12
Summer	*Pinus sylvestris*	28.0200	32.77	9.18	91.81	18.84	72.97	1.15	0.74
Winter	*Malus domestica*	37.7814	31.35	11.84	118.44	25.94	92.51	0.69	0.11
Summer	*Malus domestica*	71.8000	32.70	23.48	234.79	48.32	186.46	0.60	0.12
Summer	*Abies alba*	65.9000	41.87	27.59	275.92	38.31	237.62	1.47	0.15
Winter	*Abies alba*	36.1259	44.41	16.04	160.45	20.08	140.37	0.39	0.09
Winter	*Quercus robur*	40.9372	39.86	16.32	163.19	24.62	138.57	0.78	0.10
Summer	*Quercus robur*	69.0700	33.44	23.10	231.00	45.97	185.03	3.38	0.21
Winter	*Ulmus carpinifolia*	54.4253	40.05	21.80	217.98	32.63	185.36	1.30	0.49
Summer	*Ulmus carpinifolia*	59.6600	40.07	23.91	239.08	35.75	203.33	0.29	0.08

The viscotoxin content was done according to the methodology described by Schaller et al. 1998 (https://doi.org/10.1055/s-2006-957553).
